# Progesterone levels predict pregnancy outcomes in individuals with fallopian tube associated infertility

**DOI:** 10.1186/s12884-020-03495-6

**Published:** 2021-01-06

**Authors:** Wenjia Bo, Ning Zhang, Ling Wang, Ying Guo, Haicui Wu

**Affiliations:** 1 Shandong University of Traditional Chinese Medicine, No. 4655, University Road, University Science and Technology Park, Changqing District, Shandong 250355 Jinan, China; 2 Hospital & Institute of Obstetrics and Gynecology Laboratory for Reproductive Immunology , Shanghai Medical College Fudan University , 200433 Shanghai, China; 3 The Academy of Integrative Medicine , Fudan University , 200433 Shanghai, China; 4 Shanghai Key Laboratory of Female Reproductive Endocrine-related Diseases , Shanghai, China; 5 Affiliated Hospital of Shandong University of Traditional Chinese Medicine , 250011 Jinan, China

**Keywords:** Progesterone, Fallopian tube factor, Fresh embryo transfer, Live birth rates

## Abstract

**Background:**

This study aimed at determining the predictive value of human chorionic gonadotropin and progesterone levels on pregnancy outcomes in patients receiving *in vitro* fertilization due to fallopian tube associated infertility.

**Methods:**

We retrospectively analyzed the clinical data of 854 cycles due to fallopian tube associated infertility *in vitro* fertilization fresh embryo transfer. The clinical data had been collected from January 2010 to December 2018 and was divided into 7 groups depending on the progesterone level on human chorionic gonadotropin administration day. Live birth rates and observation trends were calculated. The receiver operating characteristic curve was established to determine the optimal cutoff value for progesterone, which was used to further divide the data into 3 groups; Group 1 (progesterone ≦ 1.0 ng/ml), Group 2 (1.0 ng/ml ≤ progesterone ≤ 1.25 ng/ml), and Group 3 (progesterone ≥ 1.25 ng/ml). We then compared the ovulation results and clinical outcomes among the 3 groups.

**Results:**

There were no significant differences in age, infertility years, gonadotropin dosage, gonadotropin days, Luteinizing hormone level on human chorionic gonadotropin day, 2 pronuclear fertilization rates, clinical pregnancy rates, live birth rates, full-term birth rate, and preterm birth rates among the three groups. However body mass index (*p* = 0.001), basal luteinizing hormone (*p* = 0.034), estrogen peak (*p* < 0.001), number of oocytes obtained (*P* < 0.001) were significantly different.

**Conclusions:**

Progesterone level on human chorionic gonadotropin day does not affect the clinical pregnancy rate and live birth rates after *in vitro* fertilization. However, progesterone levels between 1.0 and 1.25 ng/ml may lead to good clinical pregnancy outcomes.

## Background

Progesterone (P) is known to play important physiological functions during the menstrual cycle and pregnancy [1]. In assisted reproductive treatment (ART), the use of P levels during the late follicular phase to predict pregnancy outcomes remains controversial. A recent study revealed that elevated P levels on the day of human chorionic gonadotropin (HCG) administration negatively affects live birth rates and are correlated with high rates of miscarriage. However, the detrimental impact of high P levels during pregnancy is not associated with endometrial receptivity [2]. A different study reported that low P levels (≤ 0.5 ng/ml) on the day of HCG administration are associated with low live birth rates(LBR)[3]. Elevated P levels on the day of oocyte maturation have also been suggested to affect embryonic quality while high P levels (> 2.0 ng/ml) before oocyte maturation have been shown to negatively impact the oocyte [4]. It has also been documented that premature luteinization does not affect *in vitro* fertilization (IVF) outcome [5]. According to the controlled ovarian stimulation protocol, the growth of multiple follicles may result in different levels of serum P in the late follicles [6]. Paulson and colleagues found that when serum P level exceeds a certain threshold, it triggers various endometrial changes that result in the pre-transformation of the endometrium [7]. During the secretory phase, embryonic development rate gets out of sync with the endometrial phase, which negatively impacts successful implantation. Serum P levels in the late follicular phases correlate with the number of follicles formed [6]. It has been suggested that ovarian reserve functions decreases with increasing serum P levels, and that too high or too low P level might lead to premature luteinization or follicle maturation [8]. The quality of the embryo is impaired, and the opening time of the endometrial implantation window is significantly shortened, which interferes with embryonic implantation and development [9]. Multiple studies have reported that in gonadotropin-releasing hormone (GnRH) downregulated cycles, premature increase in P levels on the day of HCG administration negatively correlates with IVF outcomes. It should be noted that most of these studies included patients with different causes of infertility and the transplant date as well as the ovarian response degree were different. In addition, different P threshold values were used in different studies. These factors affect the reliability of the results and the capacity of P levels to be used as predictors of pregnancy outcomes. To address these shortcomings, we performed a retrospective analysis of the potential of P levels to be used as predictors of pregnancy outcomes in patients with simple fallopian tube defects-induced infertility. All participants in the studies were normal ovarian responders and the embryos were transferred on the third day.

## Methods

### Study design

This retrospective study was performed on a cohort of participants treated at the Center for Reproductive Medicine and Genetics at Shandong Provincial Hospital of Traditional Chinese Medicine, between January 2010 and November 2018. Of the cases seen during the study period, 854 met our inclusion criteria. Ethical approval for this study was provided by the ethics committee at Shandong Provincial Hospital of Traditional Chinese Medicine.

### Patient enrollment criteria

Participating women were included only if they met the following criteria: (i) they were undergoing assisted reproduction with fresh autologous embryo transfer on day 3 following oocyte retrieval; (ii) had infertility resulting from fallopian tube factors; (iii) they were normal ovarian responders (6–19 oocytes) [10] and (iv) had follicle-stimulating hormone (FSH) levels of < 10 IU/L on the second day of menstruation. Patients were excluded from the study if their reasons for IVF were linked to male sterility, ovarian, endometriosis, genetic, uterine or idiopathic factors. Those with hydrosalpinx were also excluded.

### Controlled ovarian hyperstimulation protocols

Pituitary down-regulation was achieved by treatment with a GnRH agonist (Triptorelin®/Diphereline®) or antagonist (Ganirelix®/Cetrotide®). The choice of protocol and gonadotrophin dose were individualized to the patient’s clinical presentation and the clinician’s preference. The dosage of gonadotropins (Puregon®/Gonal®) used varied from 75 to 450 IU/day. When the dominant follicle reached ≥ 18 mm in diameter, 10,000 IU of HCG(Ovidrel®) or 0.1 mg of GnRH agonist plus 4000 IU of HCG were added to induce final maturation and the ovum picked up 36 hours later post-treatment. Serum was obtained from the participants before administration of HCG or the GnRH agonist. Progesterone levels in the serum were quantified using quantitative electro chemiluminescence immune assay “ECLIA” on an immunoassay analyzer (Beckman Coulter). Sperms were collected through masturbation prior to IVF and embryos transferred 3 days following oocyte retrieval. Embryo quality was determined in accordance with guidelines from the Society for Assisted Reproductive Technology and graded as good, fair, or poor. Luteal support was provided through vaginal P administration or intramuscular P injection. Serum HCG levels were determined 14 days after fresh embryo transfer and beta values ≥ 50 IU taken as positive indications of pregnancy. Clinically, pregnancy was defined by the presence of an intrauterine original heart beat on transvaginal ultrasound at 7 weeks of amenorrhea. Live births were defined as babies delivered after 28 weeks of gestation.

### Data Collection

Data were retrieved from ART electronic medical records. The evaluated participant characteristics included age, body mass index (BMI),duration since infertility was diagnosed and basal serum FSH levels. Additional parameters that were analyzed included the protocols used for controlled ovarian hyperstimulation,total duration of Gonadotropin (Gn), dose of Gn, peak serum estradiol level, luteinizing hormone (LH) level, P levels on the day of HCG administration, number of oocytes obtained, 2 pronuclear (PN) fertility rate, total number of embryos, number of high quality embryos, number of transferred embryos on day 3, clinical pregnancy status, number of successful live births and the number of newborns. The primary metric was the correlation between serum P levels on the day of ovulation induction during IVF and its effect on treatment outcome.

### Statistical analysis

Data was analyzed and presented as mean ± SD, frequencies or percentages. First, a normality and homogeneity of variance tests were performed. If the population distribution conformed to normality and variance was satisfied, then parametric tests were performed. Quantitative variables were compared using independent student’s t-tests while variance analyses and categorical data were compared using chi square tests. If not, the non-parametric tests were performed. Pearson correlation coefficients were calculated to establish the relationship between P and clinical parameters. Receiver operating characteristic (ROC) analysis was performed to establish the most efficient P cutoff values to discriminate between successful and unsuccessful IVF outcomes in women undergoing day 3 fresh embryo transplantation. The best cutoff values were set on the basis of an equivalent sensitivity and specificity and the highest value for area under the ROC curve (AUC). In each cohort, univariate and multivariate analysis models were used to test the preferential effect of all independent on LBR. Statistical differences were considered to be significant when p ≤ 0.05). Statistical analyses were done using SPSS statistical suite version 21 (IBM).

## Results

A total of 854 cycles were included in this study. The average age of the participants was 31.99 years old (range 20–40 years). Patients were deemed infertile based on tubal factors only. The clinical features and cycle outcomes of the enrolled cycles are shown in Table 1.

**Table 1:** Clinical features and cycle outcomes of ovarian stimulation in patients undergoing IVF cycles

Parameter	
No. of fresh cycles	854
Age(y)	31.99 ± 4.32
BMI (kg/m^2^)	23.57 ± 3.85
Percentage of primary infertility (%)	46.37 (396/854)
Duration of infertility (y)	3.39 ± 2.38
Basal FSH (IU/L)	6.80 ± 1.61
Medication protocol	
GnRH agonist (%)	92.39 (789/854)
GnRH antagonist (%)	7.61 (65/854)
Total gonadotropin (IU)	2,662 ± 1, 087
Duration of stimulation(days)	11.64 ± 2.49
Peak estradiol (pg/ml)	2,751 ± 1,165
luteinizing hormone on HCG day (IU/L)	1.8 ± 2.16
Progesterone on HCG day (ng/ml)	1.02 ± 0.42
No. of oocytes retrieved	9.86 ± 3.32
2PN fertilization rate (%)	63.78 ± 21.17
Number of available embryos	3.79 ± 2.05
Number of high quality embryos	1.33 ± 1.53
Number of transferred embryos on day 3	1.98 ± 0.39
Number of live birth babies	
2 live birth babies	94
1 live birth baby	262
No live birth baby	498

Note: values are the mean ± SD unless otherwise noted. *BMI* body mass index; *FSH* follicular stimulation hormone; *GnRH* gonadotropin releasing hormone

First, we divided the 854 cycles into seven groups depending on the level of progesterone on the day of HCG administration. Notably, P levels below 1.25 ng /ml were associated with high live birth rates,but P levels of 1.26 ng/ml to 1.5 ng/ml were correlated with low live birth rates. Progesterone levels between 1.51 ng/ml and 1.75 ng/ml were correlated with high live birth rates, but levels > 1.75 ng/ml tended to decrease live birth rates.

Table 2 shows the factors associated with P level on the day of HCG administration. It is shown that P level was not significantly correlated with age (*p* = 0.31), duration of infertility (*p* = 0.35), basal FSH (*p* = 0.79), total gonadotropin (*p* = 0.89), duration of stimulation (*p* = 0.49) and luteinizing hormone level on HCG day (*p* = 0.25). In contrast, the P level on the day of HCG administration was significantly correlated with BMI (*p* < 0.001); peak estradiol (*p* < 0.001) and number of retrieved oocytes (*p* < 0.001). P level was positively correlated with peak estradiol (Pearson Correlation Coefficients = 0.261) and number of retrieved oocytes (Pearson Correlation Coefficients = 0.158), but was negatively correlated with BMI (Pearson Correlation Coefficients =-0.160).

**Table 2:** The relationship between serum P level on the day of HCG administration and patient’s basic and clinical characteristics

Parameter	Pearson Correlation Coefficients	*P* value
Age (y)	0.035	NS
BMI (kg/m^2^)	-0.160	<0.001**
Duration of infertility (y)	-0.032	NS
Basal FSH (IU/L)	-0.012	NS
Total gonadotropin (IU)	0.005	NS
Duration of stimulation (days)	-0.024	NS
Peak estradiol (pg/ml)	0.261	<0.001**
luteinizing hormone on HCG day (IU/L)	0.039	NS
No. of oocytes retrieved	0.158	<0.001**

Note: Pearson correlation test was used to analyze the correlation between progesterone and those items

*NS *not significant

**, Correlation is significant <0.01 level

*, Correlation is significant <0.05 level

**Fig. 1:**
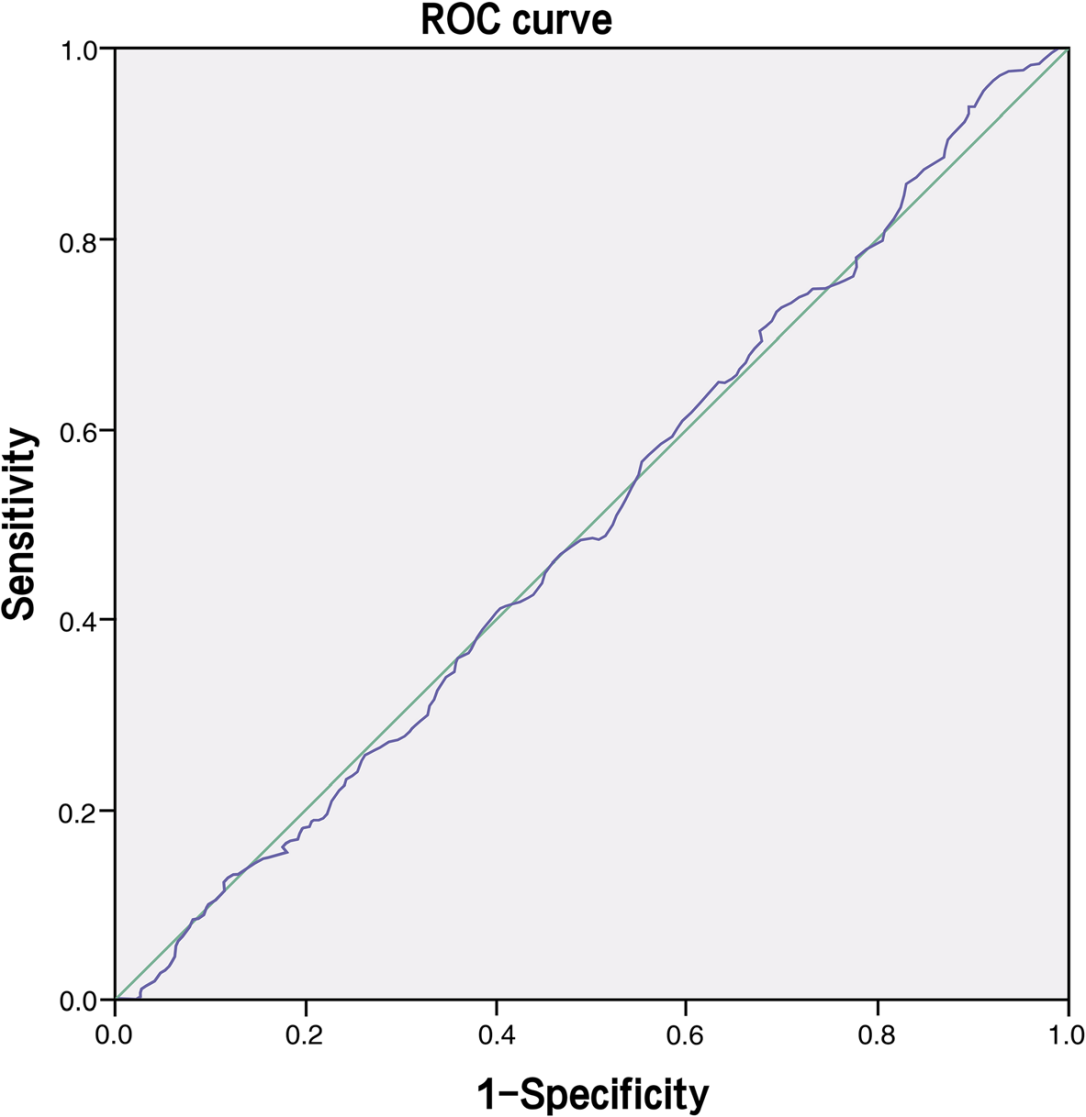
Receiver operation characteristic curve (ROC) showing the correlation between progesterone levels and live birth rate

**Fig. 2:**
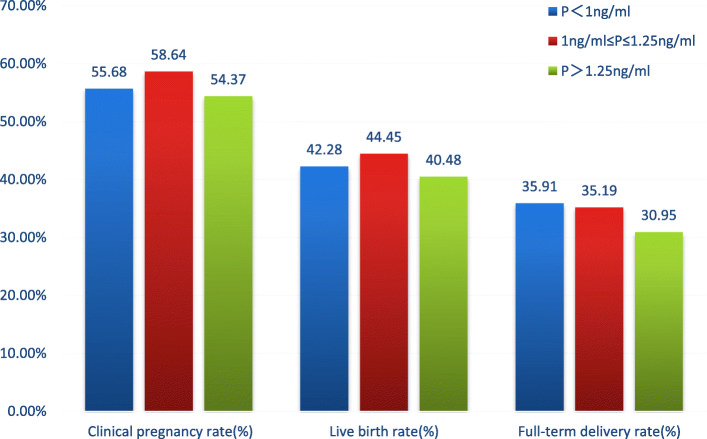
Comparison of clinical pregnancy rate, live birth rate, and full-term delivery rate among the three groups Note: P1 = 0.690 among three groups for clinical pregnancy rate P2 = 0.673 among three groups for live birth rate P3 = 0.404 among three groups for full-term delivery rate

According to the ROC analysis (Fig. 1), the optimal cut off value for P level was 1.0 ng/ml,and the AUC was 0.506 (95%CI: 0.463–0.541). To further assess the effect of serum P on late follicular oocyte and clinical outcome, we divided the data sample in three groups: Group 1 (patients with *P* ≦ 1.0 ng/ml), Group 2 (patients with P between 1.0 ng/ml to 1.25 ng/ml) and Group 3 (patients with *P* ≥ 1.25 ng/ml) (Table 3). We concluded that patients’ BMI, basal FSH, peak estradiol and number of retrieved oocytes were different among these groups. However, our analysis still showed that, patients with P levels between 1.0 ng/ml and 1.25 ng/ml had higher clinical pregnancy rates and live birth rates, although not statistically significant (*p* = 0.69,0.67 > 0.05) (Table 3; Fig. 2).

**Table 3:** Comparison between: Group 1 (patients with P ≦ 1.0 ng/ml), Group 2 (patients with P between 1.0 ng/ml to 1.25 ng/ml) and Group 3 (patients with *P* ≥ 1.25 ng/ml)

	Total	Group 1	Group2	Group 3	*P*-value
Patient demographics					
N	854	440	162	252	
Age (y)	31.99 ± 4.32	31.82 ± 4.29	32.02 ± 4.12	32.25 ± 4.50	NS
BMI (kg/m^2^)	23.67 ± 3.85	24.13 ± 3.40	23.42 ± 3.43	23.67 ± 3.85	0.001**^a^
Duration of infertility (y)	3.39 ± 2.38	3.44 ± 2.46	3.56 ± 2.60	3.21 ± 2.05	NS
Basal FSH (IU/L)	6.80 ± 1.61	6.85 ± 1.65	6.51 ± 1.57	6.90 ± 1.54	0.034*^b^
Treatment protocol and outcomes					
Duration of stimulation (days)	11.64 ± 2.49	11.70 ± 2.61	11.57 ± 2.48	11.58 ± 2.26	NS
Total gonadotropin (IU)	2662.23 ± 1087.50	2668.16 ± 1140.31	2629.49 ± 1108.43	2672.92 ± 977.73	NS
Peak estradiol (pg/ml)	2751.16 ± 1165.52	2527.78 ± 1086.78	2872.97 ± 1107.16	3062.86 ± 1252.98	< 0.001**^a^
luteinizing hormone on HCG day (IU/L)	1.80 ± 2.16	1.72 ± 2.33	1.91 ± 1.50	1.88 ± 2.20	NS
No.of oocytes retrieved	9.86 ± 3.32	9.45 ± 3.30	9.70 ± 3.15	10.66 ± 3.34	< 0.001**^c^
2PN fertilization rate (%)	63.19 ± 23.57	66.32 ± 20.32	63.18 ± 20.93	63.78 ± 21.16	NS
High quality embryo rate (%)	29.66 ± 28.57	29.79 ± 27.95	29.51 ± 29.13	29.54 ± 29.38	NS
Clinical pregnancy rate (%)	55.86(477/854)	55.68(245/440)	58.64(95/162)	54.37(137/252)	NS
Live birth rate (%)	42.04(359/854)	42.28(186/440)	44.45(72/162)	40.08(101/252)	NS
Premature rate (%)	7.73(66/854)	6.36(28/440)	9.26(15/162)	9.13(23/252)	NS
Full-term delivery (%)	34.31(293/854)	35.91(158/440)	35.19(57/162)	30.95(78/252)	NS

Note: If it is measurement data, we use analysis of variance to test; if it is count data, Chi-square test was used to test. All tests between the three groups are performed first, and if the difference between the three groups is significant, then pairwise comparisons are made

*NS* not significant

**, Correlation is significant <0.01 level

*, Correlation is significant <0.05 level

^a^, *P*-values were <0.05 in any two groups among three groups, except in the normal-versus-high group

^b^, *P*-values were <0.05 in any two groups among three groups, except in the low-versus-high group

^c^, *P*-values were<0.05 in any two groups among three groups, except in the low-versus-normal group

## Discussion

In recent years, multiple studies have proposed the potential use of P levels on the day of HCG administration as a predictor of pregnancy outcomes [11]. Some studies have shown that high P levels during daily HCG administration may adversely impact pregnancy outcomes. It has been reported that too high or too low P levels can adversely affect live birth rates. A 2013 meta-analysis based on 60,000 IVF cycles revealed that elevated daily P levels following HCG administration significantly lowers pregnancy rates upon GnRH agonist and antagonist treatment in the fresh cycle [12]. It is postulated that elevated follicular-phase P concentration produced by ovarian stimulation-induced multiple follicle growth may contribute to changes in the endometrium, leading to embryo–endometrial asynchrony [13]. A study by Yding and colleagues, analyzing 475 patients undergoing IVF assisted pregnancy, found the daily P levels on HCG administration did not affect pregnancy rates, and that elevated P levels correlated with the number of obtained oocytes. To further investigate the effect of HCG P levels on pregnancy outcomes, we selected IVF cases for infertility caused by fallopian tube factors and performed embryonic transfer on the third day after oocyte retrieval. Our study revealed that when P levels are below the range of 1.0-1.25 ng/ml, the rate of live births increases with rising P levels,indicating that a certain level of P is required for successful pregnancy. When P levels exceed this range, the LBR first drops. A follow up of the three groups revealed that the age, duration of infertility, Gn dosage, Gn days, daily HCG levels, daily LH levels, 2PN fertilization rate, clinical pregnancy rates (CPR), LBR, full-term yield and preterm birth rates were not significantly different between the groups. However BMI, basal LH levels, estrogen peak levels and number of oocytes obtained were found to significantly differ between the groups. The numbers of obtained oocytes were highest in the high P group relative to the other two groups, suggesting that higher P levels may indicate the number of resulting oocytes. These observations are in agreement with previous reports showing that elevated P levels are significantly correlated with the number of oocytes retrieved, which is in turn associated with successful IVF outcomes [14]. The rates of 2PN fertilization and quality embryos were also observed to be higher in the group with elevated P levels, although the differences were not statistically significant. It has previously been reported that post-HCG P levels are positively associated with the number of retrieved oocytes and this does not affect oocyte or embryo quality [16]. Other studies have focused on the effect of P on the embryonic stage. It has been demonstrated that when P levels exceed 1.5 ng/ml, clinically defined pregnancy rates decrease. However, similar findings were not observed following transfer at the blastocyst stage [15]. These observations suggest that controlling P levels in patients with low late follicular P levels may improve IVF outcomes [16]. Therefore, limiting the total dose of FSH administered might be beneficial [17]. For example, previous randomized trials have established that late follicular replacement of daily FSH with low-dose HCG achieves effects that are comparable to those of P receptor(PR) [18, 19] without the detrimental effects of late follicular P elevation [18]. In this study, we found that P levels ranging between 1.0 and 1.25 ng/ml exhibited better clinical pregnancy and live birth rates. Additionally, we found that HCG P levels negatively correlate with BMI and positively correlate with E2 and the number of oocytes. These findings imply that BMI, in such clinical contexts may predict HCG P levels. Many published studies have relied on different P level thresholds. Typically, when these thresholds are surpassed, the embryos are collected for freezing. Indeed, no study has demonstrated any deleterious effects of P on frozen embryo transfer [20] .Overall, HCG daily P levels have limitations as predictors of IVF outcomes. There are reports suggesting that progesterone/oocyte ratio should be considered as a tool for the prediction of IVF outcomes in reference to serum P levels alone. However, more evidence from randomized studies is required to support this conclusion [21].

In this retrospective study, we analyzed the value of HCG daily P levels to predict the success of IVF in patients with fallopian tube complications. P levels within 1.0 ng/ml to 1.25 ng/ml were associated with higher rates of clinical pregnancy and live births in the 854 cycles. Although the observed differences are not statistically significant, we contend that they can be used as a clinical references.

## Conclusions

Due to fallopian tube factors, patients undergoing fresh autologous embryo transfer on day 3 following oocyte retrieval, their P level on HCG administration day does not affect the CPR and live birth rates after IVF. However, patients with P level between 1.0 ng/ml and 1.25 ng/ml had higher CPR and LBR, although not statistically significant (*p* = 0.69, 0.67, respectively).Therefore, determining P level on HCG day may have certain guiding significance for embryonic transfer.

## Data Availability

The datasets used and/or analyzed in this study are available from the corresponding author on reasonable request.

## Abbreviations

P: Progesterone
HCG: Human chorionic gonadotropin
IVF: In vitro fertilization
ROC: Receiver operating characteristic
Gn: Gonadotropin
LH: Luteinizing hormone
PN: Pronuclear
GnRH: Gonadotropin-releasing hormone
ART: Assisted reproduction technology
BMI: Body mass index
FSH: Follicle-stimulating hormone
LBR: Live birth rate
CPR: Clinical pregnancy rate
